# Large-Scale SNP Discovery and Genotyping for Constructing a High-Density Genetic Map of Tea Plant Using Specific-Locus Amplified Fragment Sequencing (SLAF-seq)

**DOI:** 10.1371/journal.pone.0128798

**Published:** 2015-06-02

**Authors:** Jian-Qiang Ma, Long Huang, Chun-Lei Ma, Ji-Qiang Jin, Chun-Fang Li, Rong-Kai Wang, Hong-Kun Zheng, Ming-Zhe Yao, Liang Chen

**Affiliations:** 1 National Center for Tea Improvement, Tea Research Institute, Chinese Academy of Agricultural Sciences, Hangzhou, Zhejiang, China; 2 Key Laboratory of Tea Biology and Resources Utilization, Ministry of Agriculture, Hangzhou, Zhejiang, China; 3 Biomarker Technologies Co Ltd, Beijing, China; USDA-ARS-SRRC, UNITED STATES

## Abstract

Genetic maps are important tools in plant genomics and breeding. The present study reports the large-scale discovery of single nucleotide polymorphisms (SNPs) for genetic map construction in tea plant. We developed a total of 6,042 valid SNP markers using specific-locus amplified fragment sequencing (SLAF-seq), and subsequently mapped them into the previous framework map. The final map contained 6,448 molecular markers, distributing on fifteen linkage groups corresponding to the number of tea plant chromosomes. The total map length was 3,965 cM, with an average inter-locus distance of 1.0 cM. This map is the first SNP-based reference map of tea plant, as well as the most saturated one developed to date. The SNP markers and map resources generated in this study provide a wealth of genetic information that can serve as a foundation for downstream genetic analyses, such as the fine mapping of quantitative trait loci (QTL), map-based cloning, marker-assisted selection, and anchoring of scaffolds to facilitate the process of whole genome sequencing projects for tea plant.

## Introduction

Tea plant (*Camellia sinensis*, 2n = 30) is one of the most important non-alcoholic beverage crops and is extensively cultivated worldwide. In 2013, the global harvest area of tea plant reached to ~4.2 million hectares, with a production of ~4.9 million metric tons [1]. Tea is a good source of many essential micronutrients and bioactive compounds, as well as an important part of the daily diet in many countries. Numerous epidemiological investigations have suggested that drinking tea is greatly beneficial for human health [2]. Recent studies have confirmed that tea components have protective effect against various diseases, such as different types of cancer, obesity, cardiovascular and neurological diseases [3]. The consumption of tea products, particularly functional food and beverage containing tea ingredients, has increased rapidly in the past years. Thus, there is an urgent need for developing superior cultivars with attractive characteristics, such as high yield, resistance and rich secondary metabolites. However, this can’t be easily achieved because tea plant is a woody and largely self-incompatible species, which has a long juvenile phase and high heterozygosity. Recently released tea plant cultivars were primarily developed using conventional breeding methods. Those breeding procedures require a concerted effort of many breeders for a period of time to develop new cultivars. Therefore, effective tools need to be developed to accelerate tea plant breeding process. Those strategies including a combined application of novel and traditional technologies, such as marker-assisted selection (MAS) [4], have extensive potential for genetic improvement of tea plant.

Genetic maps are essential for the mapping of quantitative trait loci (QTL), MAS and map-based cloning, as well as crucial tools for the assembly of genome sequence and comparative genomic analyses. During the past 20 years, several genetic maps of tea plant have been generated with various types of molecular markers, including randomly amplified polymorphic DNA (RAPD), inter-simple sequence repeats (ISSR), amplified fragment length polymorphisms (AFLP), sequence-tagged site (STS), cleaved amplified polymorphism sequences (CAPS), and simple sequence repeats (SSR) [5–14]. However, these previous studies were performed based on either a small mapping population or small number of molecular markers, resulting in relatively low-density genetic maps with a number of gaps of no marker coverage. Until now, the highest dense genetic map of tea plant consisted of 1,124 markers [11]. In consideration of the fact that tea plant has 15 pairs of chromosomes with a large genome size of ~4 Giga base pairs[15], the process of high-density genetic map construction will be pretty time-consuming using conventional low-throughput molecular marker technologies.

Single nucleotide polymorphism (SNP) is a DNA sequence variation caused by only one nucleotide. It’s now a well-considered marker choice for high-density genetic map construction due to its abundance, uniform genome distribution, and cost-effectiveness [16]. Over the past decade next-generation sequencing (NGS) technologies have accelerated the process of large-scale SNP discovery [17]. Several efficient NGS-based methods involving a combination of SNP discovery and genotyping in a single experiment have been recently developed, such as CRoPS (complexity reduction of polymorphic sequences) [18], RAD-seq (restriction site-associated DNA sequencing) [19], GBS (genotyping-by-sequencing) [20], SBG (sequence-based genotyping) [21], and SLAF-seq (specific-locus amplified fragment sequencing) [22]. These methods firstly use specific restriction enzymes to construct reduced representation library (RRL) of genomic DNA, and which is subsequently sequenced for SNP identification and genotyping by NGS technologies. Through complexity reduction, these methods can be used for the genotyping of large genomes without the need of prior genomic information. Thus, these methods have been widely employed to facilitate the construction of high-density genetic maps in many species [23–25].

In a previous study, we have constructed a framework genetic map of tea plant using an F_1_ full-sib family [14]. However, this map showed a low map density consisting of only 406 SSR markers. Here, we used the mapping population composed of 148 F_1_ individuals randomly selected from the same full-sib family for SNP discovery and genotyping based on SLAF-seq. Finally, we constructed the first high-density genetic map of tea plant using 6448 molecular markers.

## Materials and Methods

### Plant materials and DNA extraction

An F_1_ mapping population was derived from an inter-varietal cross between *C*. *sinensis* var. *sinensis* cv. Yingshuang (‘YS’) and *C*. *sinensis* var. *pubilimba* cv. Beiyue Danzhu (‘BD’), using ‘YS’ as the female parent. Plants were grown at the Experimental Station of Tea Research Institute of the Chinese Academy of Agricultural Sciences in Hangzhou, Zhejiang, China. Details were previously described by Ma et al. [14]. Tender leaf and bud tissues were collected from each of the 148 F_1_ individuals and its parents for DNA extraction, following a CTAB method described by Dellaporta et al. [26].

### SLAF library preparation and sequencing

The procedures of SLAF library preparation and sequencing were performed as described previously [22], with minor modifications. In short, a pre-design experiment was first performed for the determination of the optimal conditions of library construction for tea plant. Based on the results of the pre-design experiment, SLAF libraries were constructed as follow. Genomic DNA from each parent and F_1_ progeny was incubated with *Mse*I (New England Biolabs, NEB), T4 DNA ligase (NEB), ATP (NEB), and *Mse*I adapter at 37°C. Restriction-ligation reactions were then heat-inactivated at 65°C, followed by digestion using *Eco*RI and NlaIII at 37°C. PCR amplification was performed using diluted restriction-ligation mixture, dNTP, *Taq* DNA polymerase (NEB) and *Mse*I primer containing barcode 1. After purification (E.Z.N.A. Cycle Pure Kit, Omega), the PCR products were pooled and incubated at 37°C with *Mse*I, T4 DNA ligase, ATP and Solexa adapter (Illumina). Then, the pooled samples were purified using a Quick Spin column (Qiagen), and separated on a 2% agarose gel. DNA fragments from 400 to 450 bp (SLAFs) were excised and isolated using a Gel Extraction Kit (Qiagen), and were then subjected to PCR amplification with Phusion Master Mix (NEB) and Solexa amplification primer mix (Illumina) to add barcode 2 according to the Illumina sample preparation guide. PCR products were gel purified, and SLAFs of 400–450 bp in size were isolated and diluted for pair-end sequencing (2×50 bp) using the Illumina HiSeq 2000 platform (Illumina) at Biomarker Technologies Co Ltd in Beijing. The raw sequence data sets have been submitted to NIH Sequence Read Archive, the accession number was SRX999833.

### SLAF-seq data analysis

SNP identification and genotyping were performed using the method as described by Sun et al. [22], with a few modifications. In brief, all of the pair-end reads with clear index information were grouped into clusters of identical sequences (SLAF tags) based on sequence similarity detected by one-to-one alignment using BLAT [27]. Then nearly identical tags were assigned to one group, which was considered as one SLAF locus. Alleles in each locus were subsequently defined using the MAF evaluation. Because of markedly lower MAF values of genotypes of tags with sequence errors, those tags were distinguished and corrected to the most similar true genotypes to improve data efficiency. For tea plant is a diploid species, one locus can contain at most four genotypes, thus SLAFs with more than four tags were discarded as repetitive SLAFs, and those with two, three, and four tags were identified as polymorphic SLAFs. In each of polymorphic SLAF locus, mismatches on the sequence of the same SLAF tag were considered as a haplotype and regarded as one potential SNP marker, while mismatches among different F_1_ individuals and the parents were genotyped and regarded as multiple alleles. To increase the accuracy, SNPs with low reads coverage and genotypes integrity in the F_1_ population were scrubbed from the dataset, and only those showing more than 4-fold reads coverage and 85% integrity were used for subsequent linkage mapping.

### Genetic map construction

The high-quality SNP markers and a set of 406 SSR markers previously mapped in this population [22] were used for genetic map construction. Linkage grouping, marker ordering, error genotyping correction and map evaluation were performed using the HighMap method as described by Liu et al. [28], with minor modifications. Briefly, markers were first clustered into linkage groups (LGs) using the single-linkage clustering algorithm based on a pair-wise modified independence LOD score as distance metric. Allelic segregation ratios were calculated for each marker by Chi-square tests. Markers were considered distorted at a level of significance of *P* < 0.05. To minimize the influence of distorted markers, a core linkage map was constructed first using Mendelian markers. Afterwards, distorted markers were separately inserted in the existing map. An iterative process of marker ordering and error genotype correction was performed to ensure the accuracy of marker order and map distances. A combination of statistic techniques, spatial sampling, Gibbs sampling and simulated annealing was used to order markers and to estimate their mutual genetic distances. The SMOOTH algorithm was used to correct genotyping errors. The LGs were assigned using SSR loci to ensure consistency with the framework map [22]. Marker pairs with zero recombination on each LG were considered to belong to the same genetic bin.

## Results

### SLAF-seq

**Fig 1:**
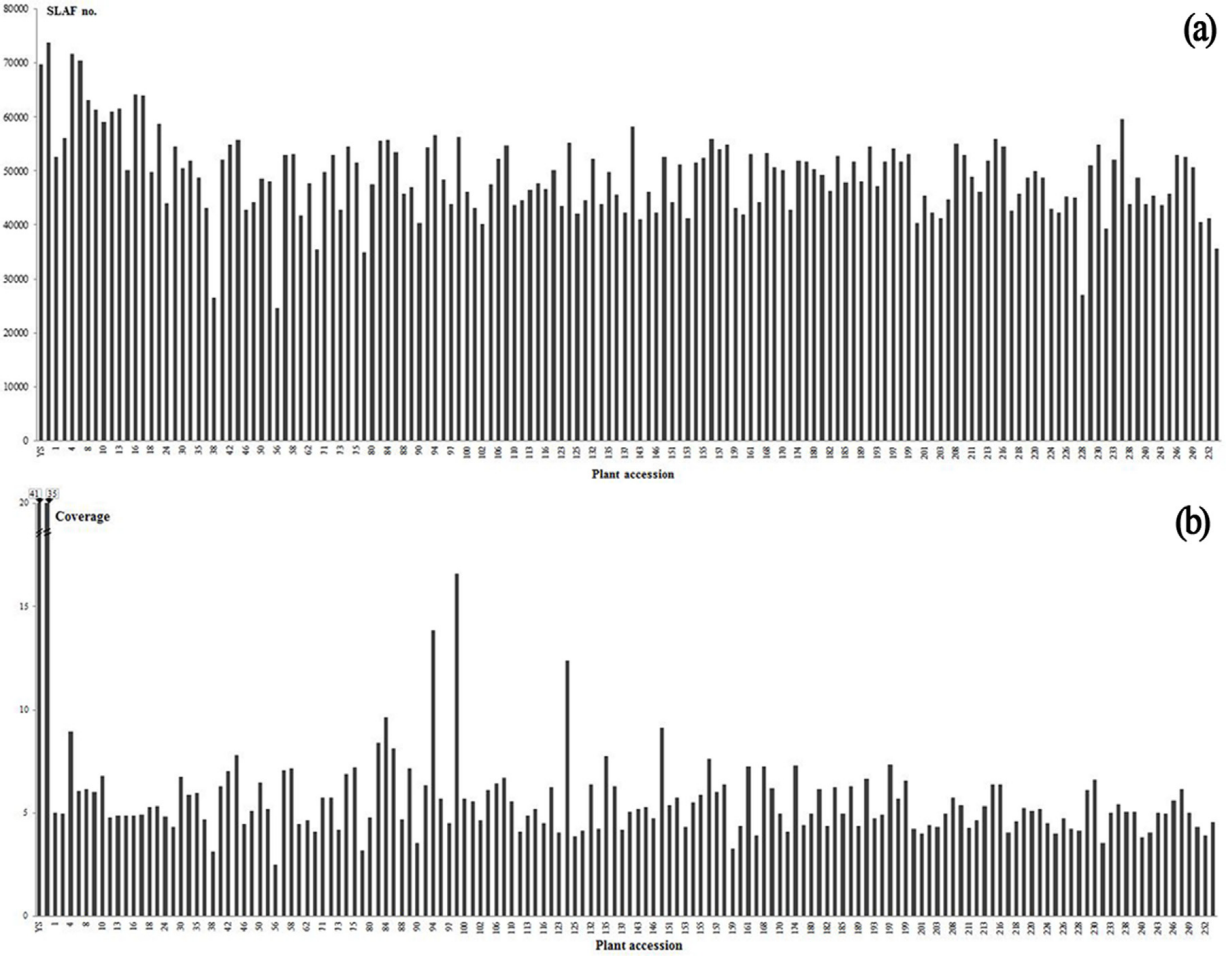
Number and coverage of high-quality SLAFs for each of the mapping parents and their offspring. The horizontal axis in both (a) and (b) indicates the plant accession, composed of the maternal parent ‘YS’, the paternal parent ‘BD’ and 148 F_1_ individuals; the vertical axis indicates the number and coverage of SLAFs in (a) and (b), respectively.

Massively high-throughput sequencing of SLAF libraries generated a total of 129,171,964 pair-end reads. Among them, the high-quality bases (*Q* score > 20) ratio was 85.4% and guanine-cytosine (GC) content was 44.3%. After sequence alignment and clustering (for details see Materials and Methods), 47,225,736 high-quality reads were assigned to 101,091 SLAF loci, of which 69,790 and 73,837 were detected in the maternal and paternal parents, respectively (Fig 1). The reads numbers for SLAFs were 2,832,781 and 2,597,955 in the maternal and paternal parents, respectively, indicating an average reads coverage of ~41 and ~35-fold for each SLAF. In the F_1_ population, the number of SLAFs per individual ranged from 24,543 to 71,697 with an average of 48,952. The reads numbers varied from 196,664 to 2,071,210 with an average of 772,669. The reads coverage was from ~3 to ~17-fold with an average of ~6-fold at the population level. Among these high-quality SLAFs, 25,014 were polymorphic, indicating an average 30.4% polymorphism rate (Table 1).

**Table 1:** Results of SLAF mining obtained from reads clustering.

Type	Reads no.	SLAF no.	Coverage	Ratio (%)
Polymorphism SLAF	14,360,532	25,014	574	30.4
Non polymorphism SLAF	32,865,204	76,077	432	69.6
Total	47,225,736	101,091	467	100.0

### SNP markers

**Fig 2:**
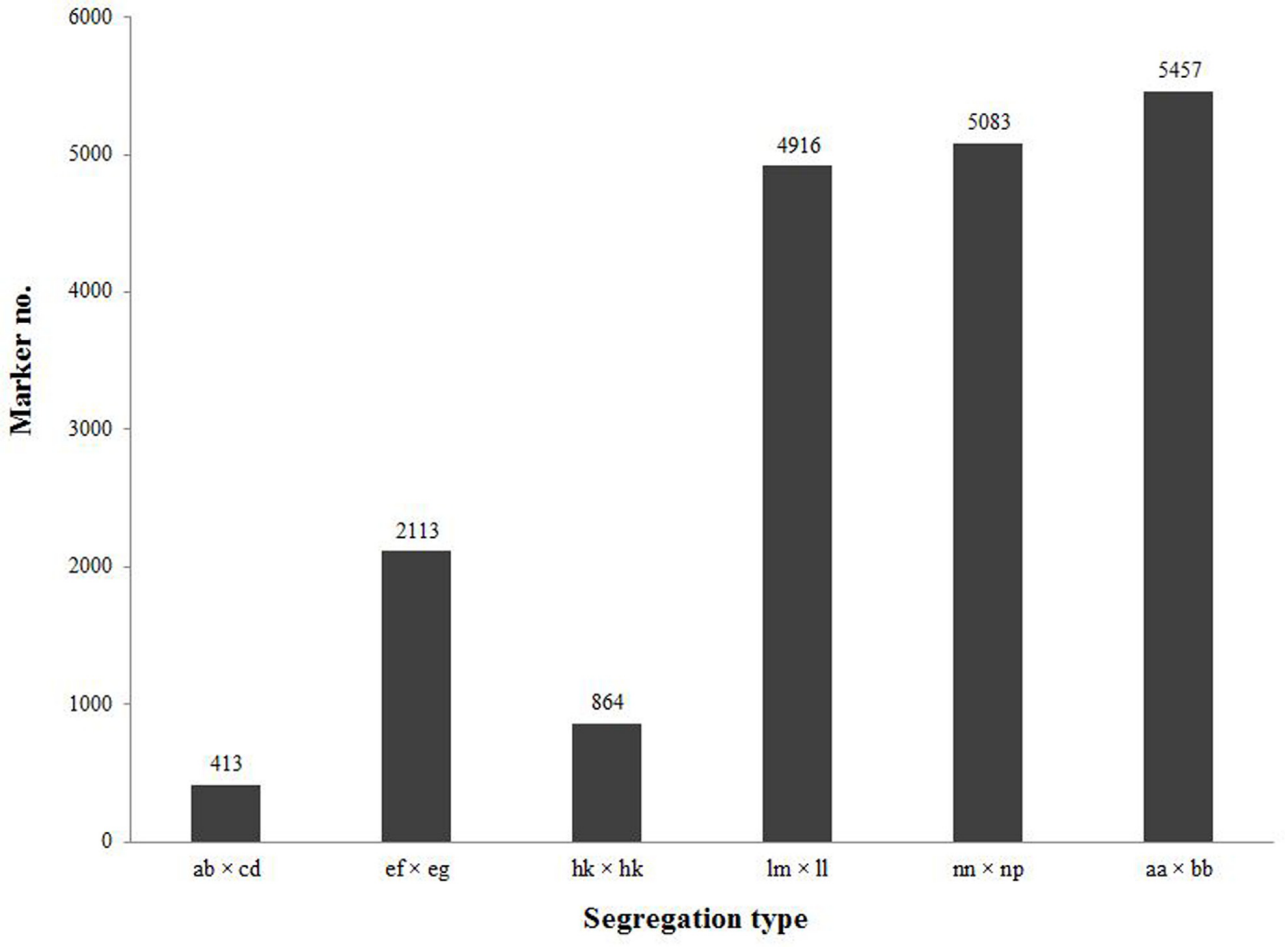
Number of SNPs for six segregation types.

After genotyping analysis and filtering out the SLAFs lacking parent information (for details see Materials and Methods), a total of 18,846 SNPs were detected and classified into six segregation types (Fig 2). The low-quality SNPs and SNPs with no segregation information in the F_1_ population (segregation type aa × bb) were discarded. This resulted in a total of 6,042 valid SNP markers with segregation type of ab × cd, ef × eg, hk × hk, lm × ll and nn × np (S1 Table). These high-quality SNPs had an average reads coverage of ~74-fold in the parents, and ~7-fold in the F_1_ progeny. The genotypes integrity of each marker among the F_1_ population was more than 99% on average. For the 6,042 SNP loci, 66.7% was heterozygous in the maternal parent, 60.3% in the paternal parent, and it ranged from 46.4% to 72.7% in F_1_ individuals.

### Genetic map

To ensure consistency with the framework map, 406 SSR markers from that map were combined with the newly developed SNP markers for use in this study. Thus, a total of 6,448 markers were used for genetic map construction. After linkage analysis, all of these markers were mapped onto the genetic map, distributing among 3,973 loci of 15 linkage groups (S1 File). Approximately 60.6% (3,905) mapped markers were grouped into bins, and 2,543 were singletons (Table 2). The average number of markers per bin was 2.7 with a median value of 2. The final map covered a total length of 3,965 cM, with an average inter-locus distance of 1.0 cM. Individually, the length of LGs ranged from 180.2 cM (LG15) to 402.2 cM (LG03). The marker numbers on each LG varied from 297 (LG09) to 599 (LG01). LG04 and LG10 had the highest marker density with an average inter-locus distance of 0.8 cM, whereas LG01 was the lowest and had a map density of 1.3 cM. The degree of linkage between adjacent loci among the LGs was presented as ‘Gap < 5 cM’, which ranged from 97.2% to 99.7% with an average of 99.2%. The largest gap of no marker coverage on this map was located in LG07 with a length of 13.2 cM.

**Table 2:** Summary of linkage groups.

Linkage group	Loci no.			Markers no.			Size (cM)	Density (cM)	^#^ Gaps <5 cM (%)	Max gap (cM)
	* Bin (Marker)	Singleton	Total	SSR	SNP	Total				
LG01	156 (436)	163	319	43	556	599	401.6	1.3	97.2	6.0
LG02	85 (216)	154	239	31	339	370	251.7	1.1	99.6	5.0
LG03	89 (210)	377	466	40	547	587	402.2	0.9	99.4	6.3
LG04	89 (251)	202	291	20	433	453	240.9	0.8	99.3	8.2
LG05	114 (327)	117	231	24	420	444	267.3	1.2	98.7	7.1
LG06	77 (207)	242	319	28	421	449	296.1	0.9	99.1	6.1
LG07	69 (172)	177	246	20	329	349	220.6	0.9	98.8	13.2
LG08	149 (458)	128	277	32	554	586	309.9	1.1	98.2	7.7
LG09	80 (216)	81	161	30	267	297	189.3	1.2	99.4	4.0
LG10	107 (277)	214	321	26	465	491	248.5	0.8	99.7	4.4
LG11	98 (249)	116	214	17	348	365	255.3	1.2	98.6	6.5
LG12	66 (163)	234	300	26	371	397	279.8	0.9	98.7	7.0
LG13	81 (216)	172	253	19	369	388	235.9	0.9	98.8	6.6
LG14	92 (294)	66	158	27	333	360	185.8	1.2	98.1	7.2
LG15	78 (213)	100	178	23	290	313	180.2	1.0	98.9	5.8
Total	1430 (3905)	2543 (39.4%)	3973	406	6042 (93.7%)	6448	3965	1.0	99.2	

* Marker pairs with zero recombination on each linkage group were considered to belong to the same genetic bin.

^#^ Percentages of locus intervals where the distance between adjacent loci was smaller than 5 cM

**Fig 3:**
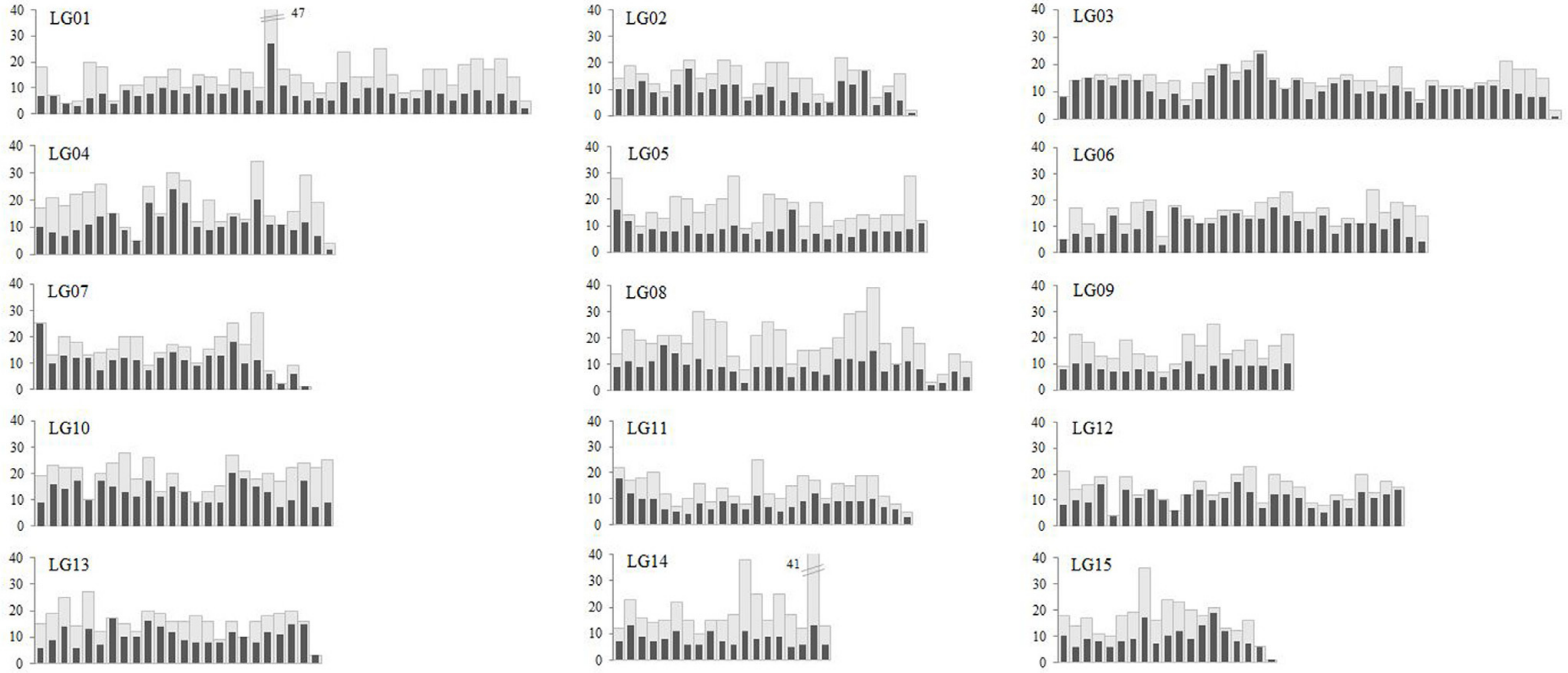
Distribution of loci and markers in intervals of 10 cM over the linkage groups. The number of loci (including bins and singletons) and all mapped markers was indicated with black and gray bars, respectively.

The distribution of markers with respect to genetic distance was analyzed for each LG. The number of loci (including bins and singletons) and all the mapped markers was counted based on 10 cM interval along the total length of each LG. As shown in Fig 3, there was few low-marker density regions scattered in the LGs. The mapped markers were almost randomly distributed along the LGs, and no significant difference between LGs was found. The average number of loci and markers within each interval per LG ranged from 8 to 13 and 14 to 20, respectively (S2 Table). In addition, the relative frequency distribution of SNP and SSR markers was also analyzed for each LG. The results were presented in S1 Fig as relative marker ratio per 10 cM interval along the LGs. SNP markers generated herein showed a tendency towards uniform distribution along the LGs. Conversely, SSR markers which were usually derived from ESTs and transcriptomes were not regular along the LGs but over-represented in some specific LG regions.

### Segregation distortion

**Fig 4:**
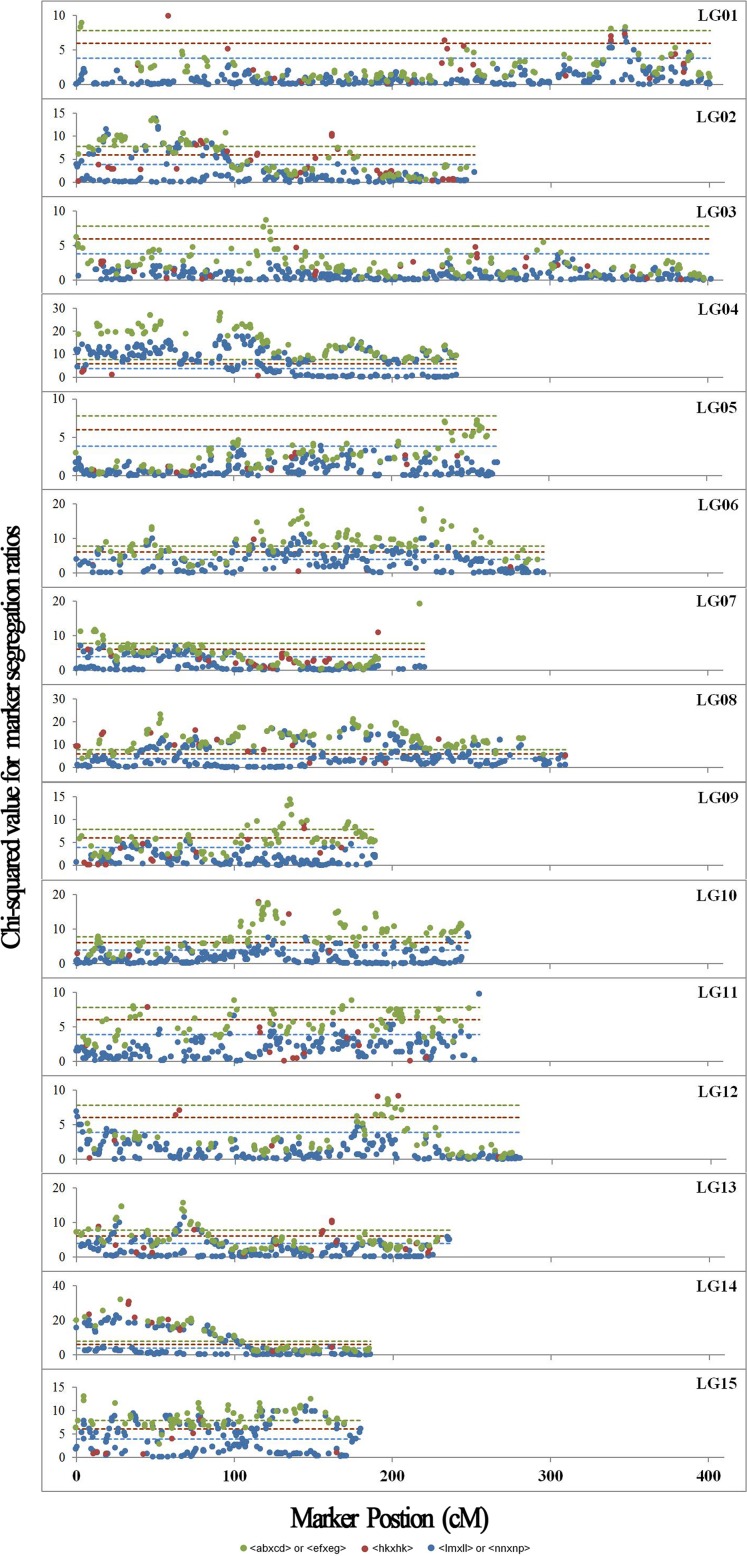
Genome-wide patterns of segregation distortion for 6448 mapped markers from different segregation types, plotted as a function of Chi-square value (y-axis) against marker position (x-axis) on each of the fifteen linkage groups. Blue, brown and green dashed lines indicate Chi-square significant values of *P* < 0.05 for marker segregation types with two, three and four genotypes, respectively.

Significant segregation distortion (*P* < 0.05) from the expected Mendelian segregation ratio was observed in all LGs (Fig 4). There were a total of 1631 (25.3%) distorted markers distributing on the map, 127 (7.8%) of which were SSRs and 1504 (92.2%) were SNPs (Table 3). This proportion was similar to that observed for all mapped markers. The frequency of distorted markers for each LG ranged from 0.34% (LG03) to 77.26% (LG04) with an average of 25.29%, while the percentage varied from 0.12% (LG03) to 21.89% (LG08) with an average of 6.67%. No significant correlation was found between the distribution of mapped markers and distorted markers. For example, the percentage of distorted markers for the two largest LGs (LG01 and LG03) was only 1.35% and 0.12%, respectively. In addition, the distorted SSRs and SNPs showed a nearly consistent tendency towards enrichment in several particular LGs, such as LG08 and LG04. All of the above suggested that the distribution of distorted markers was nonrandom across the map, and that the frequency of distorted markers on each LG was not influenced by the number of mapped markers or marker types.

**Table 3:** Summary of segregation distortion markers.

Linkage group	SSR		SNP		Total		^#^ Frequency (%)	*Segregation distortion direction			
	Number	^&^Percentage (%)	Number	^&^Percentage (%)	Number	^&^Percentage (%)		I*	II*	III*	IV*
LG01	7	5.51	15	1.00	22	1.35	3.67	9	4	1	8
LG02	10	7.87	89	5.92	99	6.07	26.76	-	88	-	11
LG03	-	-	2	0.13	2	0.12	0.34	-	2	-	-
LG04	17	13.39	333	22.14	350	21.46	77.26	227	80	-	43
LG05	1	0.79	2	0.13	3	0.18	0.68	3	-	-	-
LG06	9	7.09	180	11.97	189	11.59	42.09	94	62	-	33
LG07	10	7.87	46	3.06	56	3.43	16.05	40	12	-	4
LG08	25	19.69	332	22.07	357	21.89	60.92	226	65	-	66
LG09	6	4.72	36	2.39	42	2.58	14.14	26	8	-	8
LG10	10	7.87	98	6.52	108	6.62	22.00	40	36	-	32
LG11	-	-	34	2.26	34	2.08	9.32	29	-	-	5
LG12	-	-	20	1.33	20	1.23	5.04	12	1	-	7
LG13	3	2.36	69	4.59	72	4.41	18.56	29	32	-	11
LG14	22	17.32	101	6.72	123	7.54	34.17	96	5	-	22
LG15	7	5.51	147	9.77	154	9.44	49.20	2	141	-	11
Total	127 (7.8%)	100	1504 (92.2%)	100	1631		25.29	833	536	1	261

^&^ Calculated as the number of distorted markers per LG divided by the total number of distorted markers on the map

^#^ Calculated as the number of distorted markers divided by the total number of mapped markers per LG

* Indicating loci exhibiting skewed genotypic frequencies toward female parent (I), male parent (II), both parents (III) and heterozygotes (IV)

Among the 1,631 distorted markers, about half of them (51.1%) skewed toward the maternal parent ‘YS’, 32.9% toward the paternal parent ‘BD’, and 16.0% toward the heterozygotes and both parents (Table 3). Overall, this pattern of greater ‘YS’ allele excess among the F_1_ progeny occurred in all LGs, except LG02, LG03, LG13 and LG15. Segregation patterns of distorted markers were analyzed by using the method known as ‘allelic and zygotic segregation distortion test’ described by Li et al. [29]. The results showed that 81.5% distorted loci were caused by gametic selection, 4.9% by zygotic selection, and 10.0% by both gametic and zygotic selection (S2 File).

## Discussion

SNP markers have become increasingly popular in plant breeding due to their genome-wide abundance and biallelic nature being suitable for high-throughput automated genotyping. However, SNP discovery in complex genomes, such as tea plant, remains challenging. SLAF-seq is a recently reported method for SNP discovery and genotyping based on NGS technologies [22]. By using reduced representation strategy and deep sequencing, SLAF-seq consequently reduces sequencing costs and ensures genotyping accuracy. In contrast to conventional methods of molecular marker development and genotyping, SLAF-seq is more cost-effective and flexible. For instance, Chen et al. [30] used the SLAF-seq method in *Thinopyrum elongatum*, and a set of 7E chromosome specific molecular markers were successfully developed. A few high-density genetic maps based on SLAF-seq have been recently built in a variety of species, including sesame [25], kiwifruit [31], carp [32], soybean [33,34] and cucumber [35,36].

In this investigation, SLAF-seq was successfully employed for large-scale SNP discovery and genotyping in an F_1_ population of tea plant. Through massively parallel sequencing, we obtained ~129 M pair-end reads of 100 bp in size, which generated 101,091 high-quality SLAFs with a polymorphism rate of 30.4%. Although this is slightly lower than the 41.2% polymorphic ratio of SSRs detected previously in the same population [14], SLAF-seq easily compensates this drawback by its efficiency. From those polymorphic SLAFs, we finally developed a total of 6,042 valid SNP markers, which showed high depth of coverage in the parents (mean of ~74-fold) and F_1_ progeny (mean of ~7-fold), as well as high genotypes integrity in the mapping population with an average value of 99%. Therefore, the present study further demonstrates that SLAF-seq is an efficient method for SNP identification and genotyping in species without reference genomes, and has enormous potential for advancing tea plant genomics and breeding.

Significant differences in the total map length were observed in previously published genetic map in tea plant. For example, Hu et al. [12] developed a map with a total length of 4,482.9 cM, using a ‘TTES 19’ × ‘TTES 8’ F_1_ population and a variety of molecular markers (including 36 SSR, 3 CAPS, 1 STS, 250 AFLP, 13 ISSR and 64 RAPD). The female and male parental maps constructed by Huang et al. [8] using a ‘Qimen 4’ × ‘Chaoan Dawuye’ F_1_ population and AFLP markers, spanned a total length of 2,457.7 cM (208 AFLP) and 2,545.3 cM (200 AFLP), respectively. The genome coverage was 1,317 cM for the map reported by Taniguchi et al. [11] based on a ‘Sayamakaori’ × ‘Kana-CK17’ F_1_ population and a combination of various markers (including 441 SSR, 7 CAPS, 2 STS and 674 RAPD). The framework map that we previously generated based on the same population used herein and 406 SSR markers had a total length of 1,143.5 cM [14]. Thus, the map length variation might be expected due to the differences in the mapping population and molecular markers utilized in those studies.

The current genetic map spanned a total length of 3,965 cM, showing a significant increase in size as compared to the SSR-based framework map. The most important reason for this is likely to be the huge increase in the number of mapped markers. Because we observed that the LG size herein was strongly correlated with marker numbers (*r* ^*2*^ = 0.88) and similarly compared to the LG size of the framework map (*r* ^*2*^ = 0.76). In addition, marker type might be another reason for this change. The majority of mapped markers in the framework map were genic SSRs, which were derived from expressed regions of the genome. Thus, these SSR markers tended to distribute in some specific LG regions, leading to a relatively low degree of genome coverage and large gaps in the framework map. In contrast, SNP markers generated herein were uniformly spaced over LGs (S1 Fig). These newly mapped markers increased the global map size, either due to expansion of LGs or increasing of marker density within LGs. A total of 241 (59.7%) 10-cM intervals contained only SNP markers, meaning that these markers are filling in gaps of the framework map. Furthermore, we laid down strict criteria for the development of SNP markers and genotyping, resulting in a high level of reads coverage and integrity for each SNP locus which greatly reduced genotyping errors and consequently avoided artificial inflation of map lengths. The map accuracy was also validated by comparing marker order between the two maps, in which the order of common SSR markers was almost identical (S3 File). Altogether, these results demonstrate that our high-density genetic map seems to provide more complete coverage of the genome of tea plant.

A total of 6448 molecular markers were finally mapped onto the present map, distributing on fifteen LGs corresponding to the number of tea plant chromosomes. The average genetic distance between adjacent loci increased to 1.0 cM. To the best of our knowledge, this is the most saturated genetic map to date for tea plant. The estimated genome size of tea plant is ~4.0 Gb, which means that there is a marker every ~1,000 kb on average, although the genetic and physical distances may not correspond exactly to each other. These genome-wide molecular markers will be a useful tool for genetic diversity and association analysis [37]. In addition, almost all markers on this map are sequenced-based, providing a wealth of genomic information, which may aid in *de nove* genome assembly and comparative linkage mapping in tea plant and related *Camellia* species.

The maternal and paternal parents of the F_1_ mapping population used herein are, respectively, *C*.*sinensis* var. *sinensis* and *C*. *sinensis* var. *pubilimba*. However, the mapped markers are almost evenly distributed along the LG, and few large gaps were found on the map. It suggested that there are few chromosome rearrangements presented on the parental chromosomes homologues, which can prevent synapsis and result in recombination suppression. The number of crossing-over is generally negatively correlated with chromosome physical size [38]. In this case, the recombination rate of the genetic map, expressed as the genetic distance in centimorgans divided by the genomic length in million base pairs, is 1.0 cM/Mb on average. This is similar to the rate of genome-wide recombination in species with large genomes such as potato, maize and lettuce [38]. These results indicate that this F_1_ mapping population is suitable for the construction of high-density genetic map.

The currently available papers on genetic mapping have demonstrated that the size of mapping populations can affect the results of linkage analyses, and the increase of the number of individuals in a given population improves the precision of genetic map as well as the efficiency of QTL mapping [39,40]. Previously, we developed the reference genetic map using 183 individuals, while in this case 148 individuals were randomly selected and used. However, analysis comparing two maps found little difference in marker order (S3 File). Meanwhile, all catechins QTLs located on the reference map can be detected in the similar regions of the new map (data not shown). Therefore, the current number of individuals may be sufficient for the development of reasonably precise genetic maps and preliminary QTL identification.

Segregation distortion and clustering of the skewed loci are a ubiquitous phenomenon in many species. Previous studies indicated that the F_1_ hybrid population of tea plant usually exhibited a high level of segregation distortion [5–14]. In this investigation, a total of 1,631 (25.3%) mapped markers, including 1,504 SNPs and 127 SSRs, deviated from the Mendelian segregation patterns. This frequency was similar to that being observed previously in the same population. However, SSR markers in the current map showed a slightly higher frequency of distortion compared with their counterparts in the framework map (S3 Table). This is simply because of the difference in population sizes. In addition, we found that the distribution of skewed markers varied greatly between and within LGs on both maps. The majority (75.2%) of distorted markers mapped herein was similarly located on some particular LGs, showing a significant tendency to cluster in specific LG regions. These findings suggested that the segregation distortion in the ‘YS’ × ‘BD’ F_1_ population was caused by a genetic basis rather than an experimental artifact. Furthermore, the results of allelic and zygotic segregation distortion tests confirmed that gametic selection played a major role in contributing to the segregation distortion in the current mapping population.

In conclusion, the present study demonstrates that the NGS technologies such as SLAF-seq can be successfully used for large-scale SNP identification and genotyping in tea plant. The genetic map described in this article is the first SNP-based reference map of tea plant, as well as the most saturated one developed to date. This high-density map would serve as a foundation for QTL fine mapping, positional cloning of candidate genes, molecular breeding, and anchoring of scaffolds to facilitate the process of whole genome sequencing projects for this species.

## Supporting Information

S1 Fig Relative frequency distribution of SNP and SSR markers within 10-cM interval along the linkage groups. The ratio of SNP and SSR marker was indicated with blue and orange bars, respectively.
(TIF)

S1 File Genetic map of tea plant composed of 6448 markers. (RAR)

S2 File Results of allelic and zygotic segregation distortion test. The *P* value of maternal parental allelic test, paternal parental allelic test, and zygotic test for each distorted locus are represented as *Pa_mat*, *Pa_pat*, and *Pz*, respectively.
(XLS)

S3 File Comparison of the order of mapped SSR markers on newly developed map and framework map. The framework map is listed on the right, representing as LG01_SSR to LG15_SSR.
(RAR)

S1 Table Number of SNP and SSR markers with different segregation types used for linkage mapping. (PDF)

S2 Table The average number of loci and markers within 10 cM intervals for each linkage group. (PDF)

S3 Table Comparison of the frequency of distorted SSR markers between framework genetic map and newly developed map. The two genetic maps were constructed based on the same mapping population with a different number of F_1_ individuals. The population size was 183 and 148, respectively, for the framework map and newly developed map.
(PDF)
