# Supplementary figures and images for: Large-Scale SNP Discovery and Genotyping for Constructing a High-Density Genetic Map of Tea Plant Using Specific-Locus Amplified Fragment Sequencing (SLAF-seq)
Source: PLoS One. 2015 Jun 2;10(6):e0128798. doi: 10.1371/journal.pone.0128798 (PMC4452719; doi:10.1371/journal.pone.0128798)

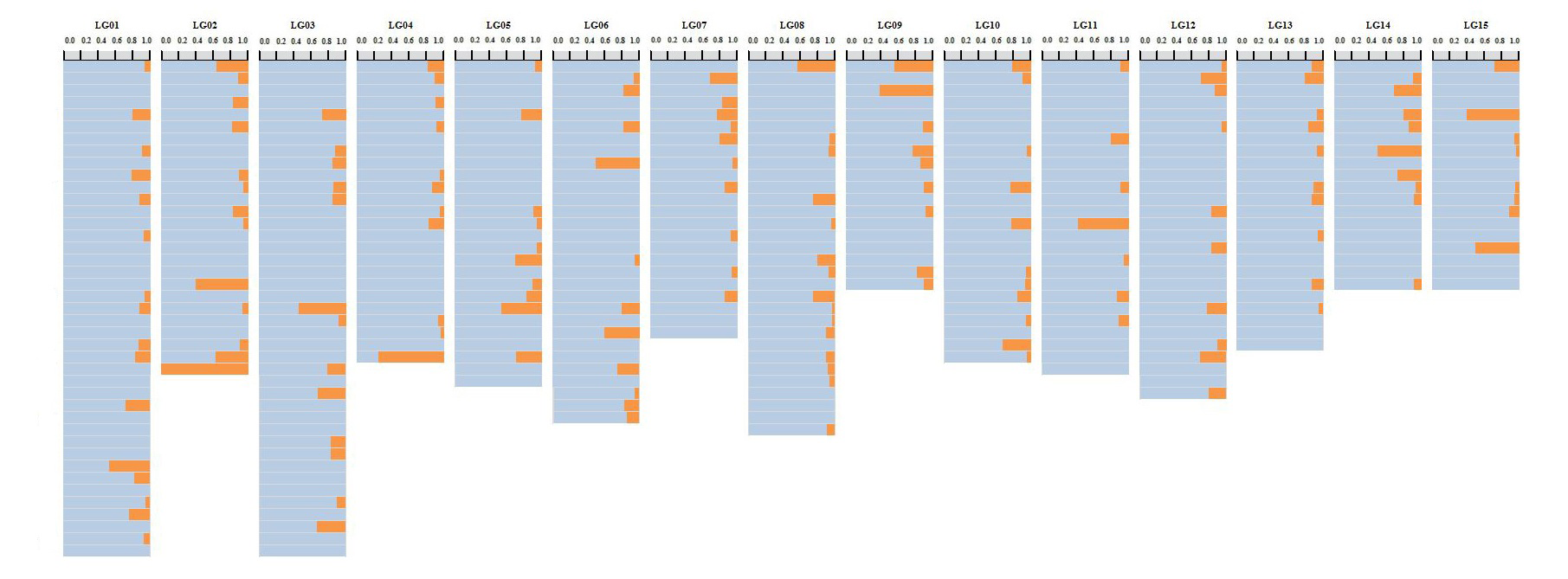

Supplement: S1 Fig — The ratio of SNP and SSR marker was indicated with blue and orange bars, respectively. (TIF) [file pone.0128798.s001.tif]
