# Supplementary material for: Large-Scale SNP Discovery and Genotyping for Constructing a High-Density Genetic Map of Tea Plant Using Specific-Locus Amplified Fragment Sequencing (SLAF-seq)
Source: PLoS One. 2015 Jun 2;10(6):e0128798. doi: 10.1371/journal.pone.0128798 (PMC4452719; doi:10.1371/journal.pone.0128798)
Supplement: S1 Table — (PDF) [file pone.0128798.s005.pdf]

S1 Table. Number of SNP and SSR markers with different segregation types used for linkage mapping.

Segregation type	SNP	SSR	Total
ab×cd	81	13	94
ef×eg	1278	118	1396
hk×hk	206	40	246
lm×ll	2492	71	2563
nn×np	1985	164	2149
Total	6042	406	6448
