# Supplementary material for: Large-Scale SNP Discovery and Genotyping for Constructing a High-Density Genetic Map of Tea Plant Using Specific-Locus Amplified Fragment Sequencing (SLAF-seq)
Source: PLoS One. 2015 Jun 2;10(6):e0128798. doi: 10.1371/journal.pone.0128798 (PMC4452719; doi:10.1371/journal.pone.0128798)
Supplement: S2 Table — (PDF) [file pone.0128798.s006.pdf]

S2 Table. The average number of loci and markers within 10 cM intervals for each linkage group.

Linkage group	Loci	Markers
LG01	8	15
LG02	9	14
LG03	11	14
LG04	12	18
LG05	9	16
LG06	11	15
LG07	11	15
LG08	9	19
LG09	8	16
LG10	13	20
LG11	8	14
LG12	11	14
LG13	11	16
LG14	8	19
LG15	8	15
Min	8	14
Max	13	20
