# Supplementary material for: Large-Scale SNP Discovery and Genotyping for Constructing a High-Density Genetic Map of Tea Plant Using Specific-Locus Amplified Fragment Sequencing (SLAF-seq)
Source: PLoS One. 2015 Jun 2;10(6):e0128798. doi: 10.1371/journal.pone.0128798 (PMC4452719; doi:10.1371/journal.pone.0128798)
Supplement: S3 Table — The two genetic maps were constructed based on the same mapping population with a different number of F1 individuals. The population size was 183 and 148, respectively, for the framework map and newly developed map. (PDF) [file pone.0128798.s007.pdf]

S3 Table. Comparison of the frequency of distorted SSR markers between published framework genetic map and newly developed map. The two genetic maps were constructed based on the same mapping population with a different number of F_1 individuals. The population size was 183 and 148, respectively, for the framework genetic map and newly developed map.

Linkage group	SSR no.	Framework genetic map			Newly developed genetic map	
		Distorted SSR no.	Frequency (%)		Distorted SSR no.	Frequency (%)
LG01	43	7	16.3		7	16.3
LG02	31	2	6.5		10	32.3
LG03	40	2	5.0		-	-
LG04	20	15	75.0		17	85.0
LG05	24	6	25.0		1	4.2
LG06	28	3	10.7		9	32.1
LG07	20	10	50.0		10	50.0
LG08	32	15	46.9		25	78.1
LG09	30	7	23.3		6	20.0
LG10	26	7	26.9		10	38.5
LG11	17	-	-		-	-
LG12	26	-	-		-	-
LG13	19	-	-		3	15.8
LG14	27	17	63.0		22	81.5
LG15	23	3	13.0		7	30.4
Total	406	94	23.2		127	31.3
